# Unobtrusive measurement of gait parameters using seismographs: An observational study

**DOI:** 10.1038/s41598-024-64508-4

**Published:** 2024-06-24

**Authors:** Michael Single, Lena C. Bruhin, Aileen C. Naef, Paul Krack, Tobias Nef, Stephan M. Gerber

**Affiliations:** 1 Gerontechnology and Rehabilitation Group, ARTORG Center for Biomedical Engineering Research, University of Bern, Bern, Switzerland; 2 Department of Neurology, Inselspital, Bern University Hospital, University of Bern, Bern, Switzerland

**Keywords:** Biomedical engineering, Health care

## Abstract

Analyzing irregularities in walking patterns helps detect human locomotion abnormalities that can signal health changes. Traditional observation-based assessments have limitations due to subjective biases and capture only a single time point. Ambient and wearable sensor technologies allow continuous and objective locomotion monitoring but face challenges due to the need for specialized expertise and user compliance. This work proposes a seismograph-based algorithm for quantifying human gait, incorporating a step extraction algorithm derived from mathematical morphologies, with the goal of achieving the accuracy of clinical reference systems. To evaluate our method, we compared the gait parameters of 50 healthy participants, as recorded by seismographs, and those obtained from reference systems (a pressure-sensitive walkway and a camera system). Participants performed four walking tests, including traversing a walkway and completing the timed up-and-go (TUG) test. In our findings, we observed linear relationships with strong positive correlations (*R*^2^ > 0.9) and tight 95% confidence intervals for all gait parameters (step time, cycle time, ambulation time, and cadence). We demonstrated that clinical gait parameters and TUG mobility test timings can be accurately derived from seismographic signals, with our method exhibiting no significant differences from established clinical reference systems.

## Introduction

Gait analysis, the systematic study of walking, serves as a medical procedure for evaluating and interpreting human locomotion^1^. Typically, this type of locomotion analysis is performed through assessments based on human observation or with the help of advanced sensor technology. Specifically, changes in locomotion patterns are examined over time to identify potential signs of abnormalities that may indicate underlying health issues or the presence of injuries. Observing such changes has been demonstrated to be valuable in detecting conditions associated with aging and neurodegenerative diseases^2–5^.

Early detection of symptoms related to these conditions enables prompt treatment of the affected patients, which helps to preserve their independence and quality of life^6^. In clinical practice, observation-based movement assessments are carried out by specialists. However, these assessments are limited by the variability and inconsistency caused by subjective biases^7^, which are a matter of concern, given that the assessments directly influence the determination of patient-specific interventions and medical treatments^8^. Furthermore, such assessments only capture a single moment in time, potentially complicating the early diagnosis of emerging diseases or the tracking of disease progression. Repeated assessments over time may be necessary to determine changes in an individual’s health status^8^. However, frequent medical visits or long-term stays at expensive healthcare facilities are currently required in standard practice, ultimately contributing to the bottleneck in performing long-term assessments^9^.

This limitation can be overcome using advanced sensor technology through the incorporation of health monitoring in smart home solutions, enabling older adults to remain in their homes and thereby reducing the need for institutional care  ^10^. Typically characterized as being either ambient or wearable, these sensor technologies are used to quantify and extend the continuity of the locomotion analysis by deriving spatiotemporal gait parameters ^11,12^. In terms of ambient sensors, pressure-sensitive walkways are a conventional clinical reference system used to measure forces while walking ^13^. However, despite their accuracy, these walkways are limited by their measurement area (because of their fixed length and width) and high acquisition costs. Motion capture systems are a popular ambient sensor technology that can be used to assess human locomotion by tracking markers placed on key anatomical features ^14,15^. These markers are used to track positions and calculate velocity, acceleration, and joint angles, but they often require expertise in their placement and data interpretation. Moreover, privacy concerns, physical challenges, and possible occlusion limit the practical application in patients’ homes ^15^. In contrast, wearable inertial measurement units, attached to the individual’s body, offer an affordable and portable solution for assessing acceleration and rotation (e.g., they can be worn on the feet to measure gait). However, they are prone to time-induced drift and need to be calibrated, and the extraction of clinical features like gait information requires algorithmic processing ^16^. Furthermore, their use in uncontrolled settings (e.g., long-term measurements at home), particularly by older adults or patients with neurodegenerative conditions, can be challenging owing to low acceptance and compliance rates, potentially making them difficult to use for longitudinal studies ^17,18^. Thus, there is a need for a robust, unintrusive, and contactless measurement system capable of monitoring human gait over prolonged periods in clinical and residential settings that requires minimal user engagement and is cost-effective.

In light of these requirements, seismographs are a promising ambient sensor technology that can overcome these challenges while offering a high degree of user compliance ^19^. These vibration-based sensors, when combined with supervised classification techniques, have been demonstrated to accurately recognize the footsteps of individuals and can even differentiate between individuals ^20,21^. Wang et al. proposed an unsupervised method based on morphological operators to extract footstep events from seismographic signals, but without calculating clinical gait parameters or comparing their derived steps to a reference system ^22^.

Despite the aforementioned limitations, the work of Wang et al. helped us develop a morphological operator complemented by spatiotemporal filters to measure clinical gait parameters accurately. The objective of this study was to present a seismograph-based methodology to quantify human gait and streamline timed gait assessments, specifically focusing on the frequently used timed up-and-go (TUG) test ^23^. We hypothesized that the accuracy of gait parameters of healthy participants measured with seismographs in a home-like environment is not significantly different from that of gait parameters measured with the reference system, namely a pressure-sensitive walkway. Furthermore, we hypothesized that TUG timings between the seismographs and the camera system were not significantly different.

## Results

### Descriptive analysis of step length and velocity

**Figure 1:**
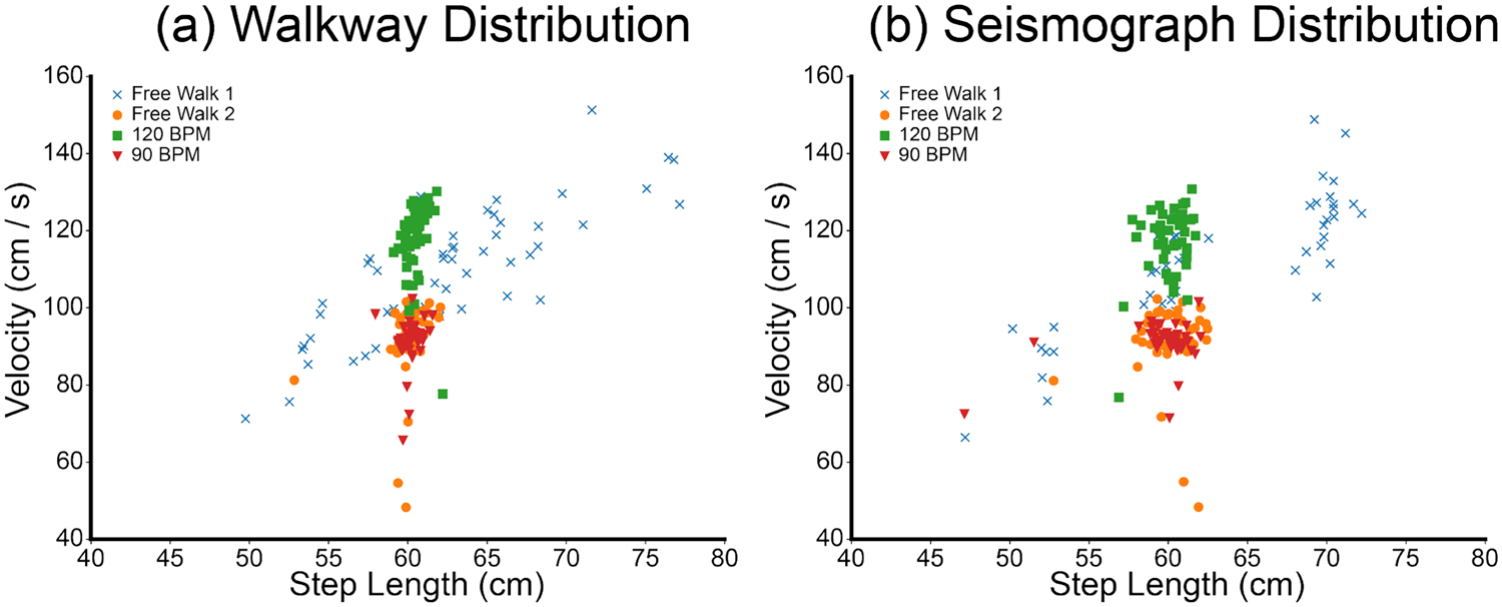
Distributions of step lengths and velocities across the different ambulation experiments. The pressure-sensitive walkway distribution is shown on the left (**a**), and the distribution generated using the seismographs is shown on the right (**b**). The blue crosses and orange circles represent free-walking data, the green rectangles represent fast-walking (120 bpm) data, and the red triangles represent normal-walking (90 bpm) data.

The distributions of step lengths and velocities showed similar characteristics for all conducted experiments (Fig. 1). The mean velocity of the pressure-sensitive walkways was 103.0 cm/s (SD = 16.6 cm/s), ranging between 48.3 and 167.0 cm/s, and the mean velocity of the seismograph was 102.9 cm/s (SD = 16.5 cm/s) ranging between 48.4 and 165.5 cm/s. The mean step length of the pressure-sensitive walkway was 60.9 cm (SD = 3.9 cm), ranging between 49.8 and 86.2 cm, and the mean step length of the seismograph was 60.7 cm (SD = 4.2 cm), ranging between 47.1 and 82.9 cm.

In this study, a total of 200 ambulation experiments were performed and recorded by 50 participants. The proposed seismograph method detected the exact number of performed steps (1577 of the 1577 steps). When employing a single seismograph-Seismograph 1, Seismograph 2, or Seismograph 3-the proposed method identified 1565, 1573, and 1567 steps out of 1577 steps, respectively. Among these ambulation experiments, the distribution of Free Walk 1 (blue crosses in Fig. 1) covered a wider, less-centered range in step length and velocity than the distribution of Free Walk 2 (orange circles in Fig. 1). Moreover, certain seismograph step length values in Free Walk 1 were either over- or underestimated compared with the pressure-sensitive walkway. The other three ambulation experiments (i.e., Free Walk 2, Swift Walk, and Normal Walk) exhibited matching distributions.

### Comparison of gait parameters between seismographs and walkway

The outcomes of the paired two-sample t-tests revealed no statistically significant difference between the gait parameters measured with the pressure-sensitive walkway and those measured with seismographs (Table 1). Furthermore, for all evaluated gait parameters, similar mean, and standard deviation values were found with small associated effect sizes (d < 0.2). Likewise, upon categorizing the measured individuals based on age (e.g., those above 40 years and those 40 years or younger), there was no significant difference in the gait analysis results obtained from both the pressure-sensitive walkway and the seismographs. Comparable outcomes were also found when different age groups were formed (detailed results are available in our repository)^24^.

**Table 1:** Paired two-sample t-tests were conducted on the gait parameters, namely step time, cycle time, ambulation time, cadence, velocity, and step length.

Gait parameter	Walkway		Seismograph		t(200)	p	d
	M	SD	M	SD			
Step Time (s)	0.604	0.092	0.603	0.092	-1.583	0.115	0.008
Cycle Time (s)	1.206	0.186	1.205	0.185	-0.556	0.579	0.003
Ambulation Time (s)	4.206	0.737	4.199	0.737	-1.528	0.128	0.011
Cadence (min^−1^)	101.230	13.780	101.378	13.620	0.893	0.373	0.011
Velocity (cm/s)	103.035	16.603	102.987	16.529	-0.168	0.867	0.003
Step Length (cm)	60.997	3.918	60.737	4.200	-1.436	0.153	0.064

No significant differences were found between the gait measurements on the pressure-sensitive walkway and the seismograph at the specified significance level (cf. determined p-values, which were always larger). The degree of freedom for all tests was 199. Furthermore, the mean and standard deviation values were comparable across both measurement devices. A small size effect was observed, as indicated by Cohen’s d statistic.

We found a significant, strong positive correlation with small standard errors for all analyzed gait parameters between the measurements obtained with seismographs and those obtained with the pressure-sensitive walkway (Table 2). The Pearson correlation coefficients were above 0.970, with the exception of step length, which exhibited a Pearson correlation coefficient of 0.804. Furthermore, the analysis revealed that for all assessed gait parameters, with the exception of step length, both the lower and upper bounds of the 95% confidence intervals were high (LL > 0.950 and UL > 0.975). Conversely, the confidence interval for step length ranged from 0.749 to 0.848, indicating a lower degree of correlation relative to the other parameters.

**Figure 2:**
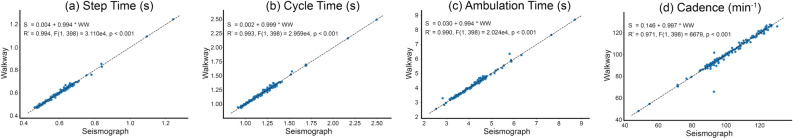
Plots of the linear relationships of gait parameters between measurements assessed using the pressure-sensitive walkway (WW) and the seismographs (S). In each plot, we provide the slope and inclination of the fitted linear model, the determination coefficients, and the F-scores. (**a**) Step Time, (**b**) Cycle Time, (**c**) Ambulation Time, and (**d**) Cadence.

The F-statistics showed significant linear relationships (p < 0.001) with slopes close to one between gait parameters measured using the seismographs and the pressure-sensitive walkway (Fig. 2). In this context, high coefficients of determination (i.e., $R^2 > 0.9$) were found for the analyzed gait parameters except for step length, which exhibited a moderate coefficient of determination ($R^2 = 0.626$), as further detailed in the supplementary material (Supplementary Material, Fig. S2).

**Figure 3:**
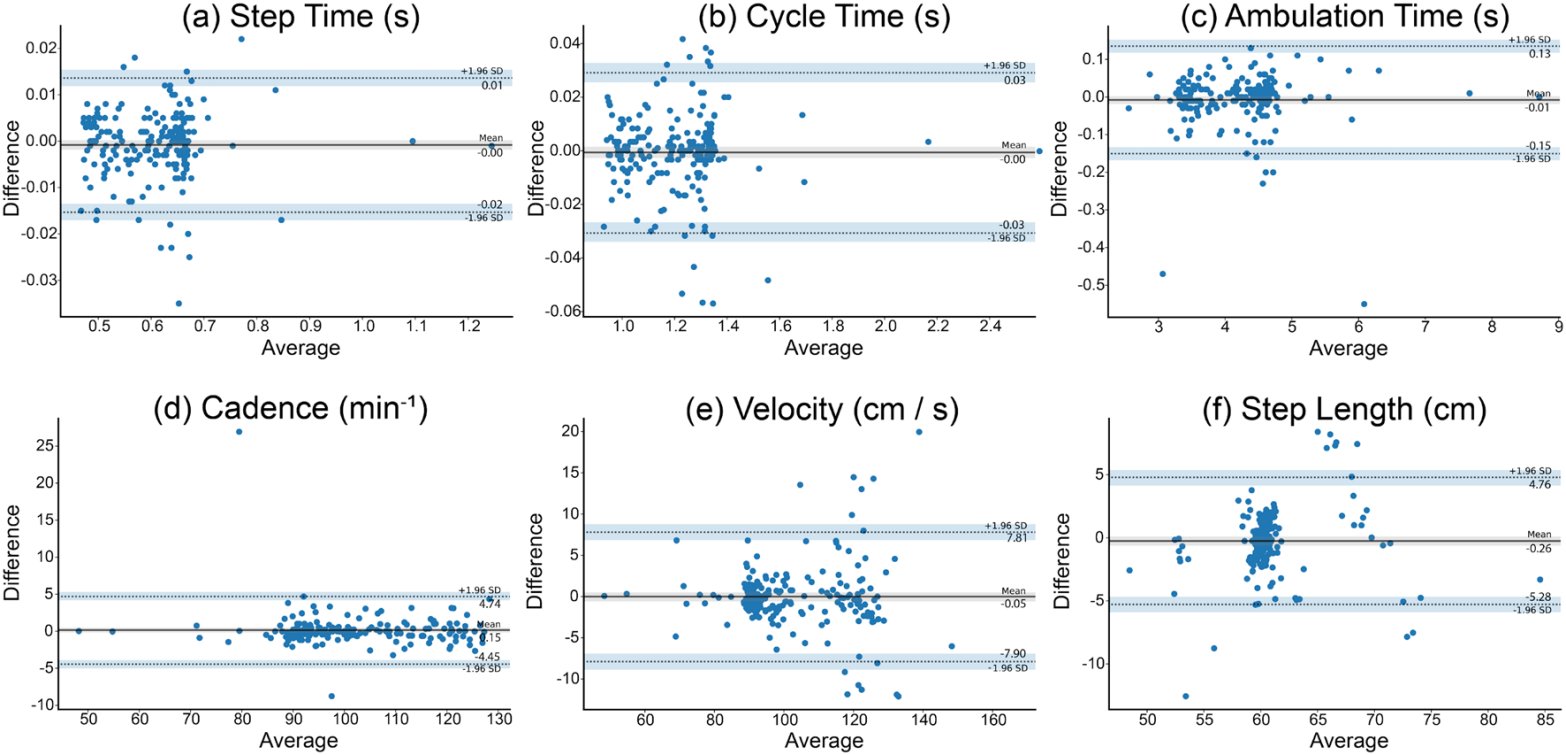
Bland-Altman plots were used to demonstrate agreement of matching gait parameters between the seismograph and the walkway measurements. No proportional bias and a close-to-zero constant bias were found. The limits of the agreements, encompassing 95% of all differences between measurements, exhibited acceptable values: The step time was between − 15.27 and 13.62 ms, the cycle time was between 30.32 and 29.13 ms, the ambulation time was between − 150.54 ms and 134.81 ms, the cadence was between − 4.45 and 4.74, the velocity was between − 7.90 and 7.81 cm/s, and the step length was between − 5.28 and 4.76 cm.

The Bland-Altman plots revealed an agreement between gait parameters measured by the pressure-sensitive walkway and the seismographs (Fig. 3). The analysis identified negligible biases, indicating an absence of proportional bias and a near-zero constant bias. Furthermore, the limits of agreement, which encompass 95% of the discrepancies between the two sets of measurements, demonstrated acceptable values with only a few outliers. Additionally, the variation in differences across various means was uniformly low and consistent.

**Table 2:** Pearson correlation statistics of simple linear regression models between seismographic and pressure-sensitive walkway measurements.

Gait parameter	*r*(200)	*SE*	95% CI		*p*
			LL	UL	
Step Time (s)	0.996	0.006	0.996	0.998	< 0.001
Cycle Time (s)	0.996	0.005	0.996	0.997	< 0.001
Ambulation Time (s)	0.995	0.007	0.994	0.996	< 0.001
Cadence (min^−1^)	0.985	0.012	0.981	0.989	< 0.001
Velocity (cm/s)	0.971	0.017	0.962	0.978	< 0.001
Step Length (cm)	0.804	0.039	0.749	0.848	< 0.001

We observed a strong positive correlation for all gait parameters. Number of walks = 200; CI = confidence interval; LL = lower limit; UL = upper limit; SE = standard error; r is the Pearson correlation coefficient.

### TUG test assessments with seismographs

A total of 50 TUG tests were recorded. The TUG test timings for the camera system ranged between 5.338 s and 10.811 s, and those for the seismographs ranged between 5.110 s and 10.670 s. There was no significant difference in measured TUG times between the camera system ($M = 8.303 s, SD = 1.250 s$) and the seismographs ($M = 8.301 s$, $SD = 1.273 s$), $t(50) = -0.038$, $p = 0.970$. We also observed a significant linear relationship between timings measured with seismographs and the camera system ($F(1, 98) = 1.045, p < 0.001$), with a high coefficient of determination ($R^2 = 0.970$) predictor. Finally, we found a strong positive correlation between the average camera system and seismograph timings ($r(50) = 0.985, p < 0.001$).

## Discussion

In this observational study, we developed a novel method for deriving clinically relevant gait parameters from seismographic measurements. In line with our first hypothesis, the study provided evidence showing that the mean values of matching gait parameters derived from seismographic and pressure-sensitive walkway measurements exhibited no significant differences. Furthermore, the findings substantiated our second hypothesis, revealing that the TUG mobility test timings determined using the seismographs did not significantly deviate from those obtained using the camera system.

Consistent with Li et al.’s findings, our results confirmed the effectiveness of integrating mathematical morphologies with CTF for isolating seismic events of interest (i.e., footsteps) while mitigating low-frequency noise in vibration signals. Compared to bandpass filtering, the CTF method exhibits greater resilience against the frequency mixing issue, where seismic signals and noise overlap in frequency bands. Additionally, CTF demonstrated great potential in diminishing low-frequency noise compared to both white and black top-hat transformations^25^.

Although damping slightly weakened the strength of measured seismic signals, the effectiveness of our step extraction algorithm remained unaffected. This robustness can be attributed to a combination of strategic approaches within our methodology. Firstly, the identification of footstep events was based exclusively on the timestamp of peaks rather than their amplitude. Secondly, peaks produced by applying the attenuation stage exhibited distinct characteristics compared to non-peaks. Lastly, we implemented aggregation strategies, including the maximum combination of seismic signals measured by the three seismographs, to enhance any diminished amplitudes, effectively counteracting potential negative effects on signal quality (Supplementary Material, Section S5). Our findings demonstrated that even with the use of just one seismograph, our algorithm was capable of successfully identifying nearly 99% of all performed steps.

The comparison between gait parameters - including step time, cycle time, ambulation time, cadence, speed, and step length - measured with seismographs and the pressure-sensitive walkway showed a linear relationship with strong positive correlations. These findings align with existing studies regarding the accuracy of temporal gait measurement^16,26,27^. Additionally, the distributions of velocity and step length obtained through the seismograph closely mirrored those obtained through the pressure-sensitive walkway. Given that such distributions have been previously identified as indicators of health changes in older adults, our results imply that seismographs could effectively function as a reliable digital tool for monitoring these health variations^28^. It is worth noting that Free Walk 2 was performed immediately after the 90bpm walk, leading to the assumption that the metronome beat impacted participants’ walking behavior. Although the Free Walk 2 distribution diverges from typical free-walking behavior, the similarity between the Seismograph and Walkway distributions indicates that the discrepancy originates from the experiment protocol rather than the measurement techniques.

Gait parameters that were computed based on the temporal detection of two consecutive steps showed a high agreement between the two systems, and thus, systematic errors in step detection did not have an impact on them and were consistent across consecutive steps. Regarding the measurement errors, the determined ranges of velocity, step time, and cadence were confirmed by referring to findings from the literature^29,30^. Therefore, the results provide evidence that the three seismographs detected steps with high consistency and reliability. These findings further demonstrate that seismograph-based gait assessments are a reasonable method to measure gait parameters in a clinically acceptable range.

In this study, the calculation of spatial gait parameters, including step and stride lengths, relied on approximations using the known length of the walkway. However, this reliance on approximation is not an inherent limitation of seismographic measurements themselves, as demonstrated by Mirshekari et al.^31^. The decision to use approximations stemmed from the fact that the accuracy of seismographic-based localization is contingent upon the sensors’ sampling rate. In our specific instance, the seismographs operate at a sampling rate of 100 Hz. This rate was found to be insufficient for achieving the necessary temporal resolution required for accurately estimating the location of seismic events of interest.

The statistical analysis conducted between the seismographs and the camera system showed a strong positive correlation and a high accuracy in assessing the timings of the TUG test. These findings indicate that the seismograph-based method could help to automate the evaluation of timed mobility tests, ultimately leading to a more objective medical assessment. Notably, an accurate assessment of these timing results was achieved without intrusive installation or calibration of the sensing devices. Considering the cost-effectiveness of seismographs, the developed method is not only promising as an ambient technology but also as a reliable solution for ongoing home monitoring of older adults^32^.

## Limitations and Outlook

First, although the linear models demonstrated high coefficients of determinations, signifying that more than 90% percent of their variations could be explained by the model, the calculated spatial gait parameter (e.g., step length) performed worse than its temporal counterpart (e.g., step time). This difference in accuracy is a direct consequence of the low sampling rate of the chosen off-the-shelf seismograph systems, necessitating a simplified heuristic approach for calculating spatial gait parameters (i.e., the approximations we used as described in Table 4). Inspired by acoustic-based localization techniques, this limitation could, however, be reduced by increasing the measuring sampling rate and then correlating the measured temporal events across different seismographs with their respective positions^33^. Consequently, the accuracy of the resulting step lengths would then solely depend on the precision of the event timestamps, but this approach would require custom-built sensor hardware.

Second, this study only used gait measurements from healthy participants, which potentially restricts its general applicability, especially considering the significant differences in gait patterns found in individuals with neurological disorders or in older adults^11^. Hence, subsequent studies should include patients who have walking impairments or belong to an older adult demographic with a risk for falling.

Third, to minimize external influences that could potentially degrade the quality of the seismic measurements obtained, the proposed step extraction algorithm required a controlled environment, ensuring that no individuals other than the individual under observation. However, when considering the application of these sensors in real-world home environments, the presence of additional individuals in close proximity to the sensors is inevitable. The capability to track the steps of multiple individuals using seismic signals was demonstrated through the deployment of an array of seismographs^34^. Although the original researchers did not delve into identifying individuals based on their unique seismic patterns, this area has gained significant interest in recent years^35–37^. Consequently, for the purpose of enabling seismograph-based gait analysis in residential settings inhabited by multiple occupants, future studies could benefit from investigating the integration of an array-like configuration of multiple sensors alongside advanced techniques for seismic-based person identification. This approach represents a promising direction for research, aiming to enhance the application of seismograph technology in everyday environments.

## Conclusion

This study presents a novel seismographic-based method that can be used to measure clinical gait parameters. We demonstrated that the gait parameters derived using our method are not significantly worse than those assessed with state-of-the-art technology (i.e., video-based motion tracking systems and pressure-sensitive walkways). Additionally, we showed that these cost-effective seismographic sensors work in home-like environments. The gait analysis algorithm is based on morphological operators that filter features of interest and simultaneously attenuate noise in the seismographic signal. This sensor technology allows researchers to autonomously determine clinical timings in ambulation assessments like the TUG test commonly used by medical professionals. Finally, these unobtrusive ambient sensors represent a promising technology with high accuracy in measuring clinical gait parameters outside of the hospital environment that could be used to conduct longitudinal studies in home-like settings.

## Methods

### Participants and setting

A convenience sample of 50 healthy individuals ranging in age from 19 to 71 years (mean, 32.86 years; SD, 11.08 years) was recruited for this observational, cross-sectional study^38^. The sample was gender-balanced, encompassing 26 women and 24 men. Participants were eligible for inclusion if they were at least 18 years of age, and exhibited no walking impairments that could affect their regular daily activities. The study protocol was explained to each participant verbally, and written informed consent was obtained prior to participation. This study was conducted over three weeks (from November to December 2021) in a home-like instrumented apartment, the NeuroTec Loft, located at the Swiss Institute for Translational and Entrepreneurial Medicine (Inselspital Bern, Switzerland)^38,39^.

This study was approved by the Ethics Committee of the Canton of Bern, Switzerland (KEK no. 2020-02771, date of approval: 18.03.2021) and conducted in accordance with the latest version of the Declaration of Helsinki.

### Experimental procedure

Each participant undertook four predefined ambulation experiments (i.e., two free walks and two walks with a metronome, one to a fast beat and one to a normal beat) by walking over the walkway (Table 3) and performing the TUG test according to its protocol (Supplementary Material, Section S4). Prior to initiating a measurement, participants were provided detailed instructions on the execution of the forthcoming ambulation. To ensure that the participants understood the experiment instructions, they were asked to do a test run before each ambulation experiment. The free-walking experiments were recorded twice and conducted at a self-regulated pace, thereby allowing participants to choose any pace that felt comfortable and natural to them. The remaining two ambulation experiments were conducted at a prespecified pace using a metronome.

**Table 3:** Three different ambulation experiments were performed by the participants under the provided instructions.

Experiment	Pace (bpm)	Metronome	Instruction
Free Walk	N/A	No	Start walking from the starting point at any pace until you reach the end of the walkway
Fast Walk	120	Yes	Listen to the metronome and walk to its beat. Start walking from the starting point until you reach the end of the walkway
Normal Walk	90	Yes	

Free walking was performed twice, and normal and fast walking were performed once. Note that the value N/A is used to express the notion of “not specified”, implying that this was chosen by the study participants.

During the measurement, participants were requested to traverse the walkway without wearing their shoes, thereby eliminating the dampening effect of footwear as a confounding variable. We did not take into account the influence of any further material-related damping effects concerning the seismic amplitudes, as preliminary measurements before our study showed distinct amplitudes associated with footstep events in the recorded seismic signals. To further reduce external influences that might have significantly affected the quality of the measured data, particularly concerning the seismic measurements, no person other than the patient being measured was in the vicinity of the sensor devices or walking around. For consistency, the starting position of every recorded walk was specified at the leftmost end of the pressure-sensitive walkway. Participants were requested not to leave the active area of the walkway at any point during the measurement to ensure the quality of the recorded data.

### Data collection systems

**Figure 4:**
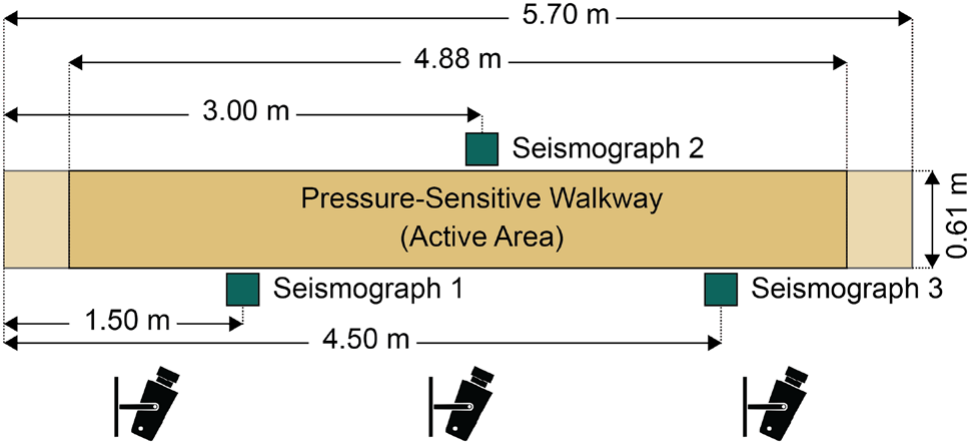
Participants were measured as they walked along the carpet from left to right. The seismographs were placed 1.5 m apart (two on the right side and one on the left side, in the direction of walking). The total length of the walkway was 5.70 m, and the active measurement length was 4.88 m. Each walk was also recorded using a camera system.

Three seismographs (RS-4D, Raspberry Shake S.A, Alto Boquete, Panama) and two reference systems, namely a pressure-sensitive walkway (GAITRite$\circledR$, CIR Systems Inc. Clifton, NJ 07012, USA) and a camera system (Qualisys QTM, Gothenburg, Sweden), were installed in the living room of the apartment to measure participant’s gaits (Fig. 4). The three seismographs were positioned on the floor along the walkway, maintaining a distance of 1.5 m between each of them. The first and last sensors were placed on the right side (in the direction of walking), while the second was situated on the left side. This sensor arrangement was used to facilitate the subsequent triangulation-based algorithmic steps. The camera system was calibrated to ensure an optimal range of angles, with each camera directed toward the walkway. The decision to incorporate a camera system alongside the pressure-sensitive walkway was motivated by the limitation of the walkway’s measurement software, which was only capable of processing complete walks (i.e., participants walking from the starting point of the walkway to its end). This was especially crucial for accurately assessing timings in TUG tests, which involved a turning point on the walkway, resulting in crashing the measurement.

The three seismographs were employed to quantify the ground motion. These sensors utilize integrated geophones designed to measure vibrations at a low natural frequency of 4.5 Hz. This makes them ideally suited for accurately measuring human gait, considering that the effective natural frequency of human walking is below 2 Hz^40^. The seismographs were configured to sample at a frequency of 100 Hz. A basic Python client-server application was developed to read the seismic samples from the RS-4D seismographs and transfer them via the network to a database using the Sensor Recording Software (SRS) system^41^. To measure spatial and temporal gait parameters, a pressure-sensitive walkway was used as a reference system. The specific walkway model employed had an active measurement length of 4.88 m and a width of 0.61 m. The data gathered from the system were sampled at a frequency of 80 Hz and analyzed using the GAITRite$\circledR$ software (version 4.89H9). The following gait parameters were obtained from the analysis: average step time, average cycle time, total ambulation time, cadence per minute, average velocity, and average step length. In addition to the pressure-sensitive reference system, a markerless motion tracking system comprising 13 Miqus video cameras (further referred to as the camera system) was used to quantify the number of steps taken by the participants and determine the duration of their walking activities. All video recordings were set to a resolution of 1920 × 1080 pixels, sampled at a frequency of 85 Hz. The camera recordings were used to determine the timestamps of events associated with the TUG test at a frame-wise precision. The timings of the video frames were extracted by using the software Adobe Premiere Pro CC 2020 (version 14.3.1).

### Gait parameter computation

**Figure 5:**
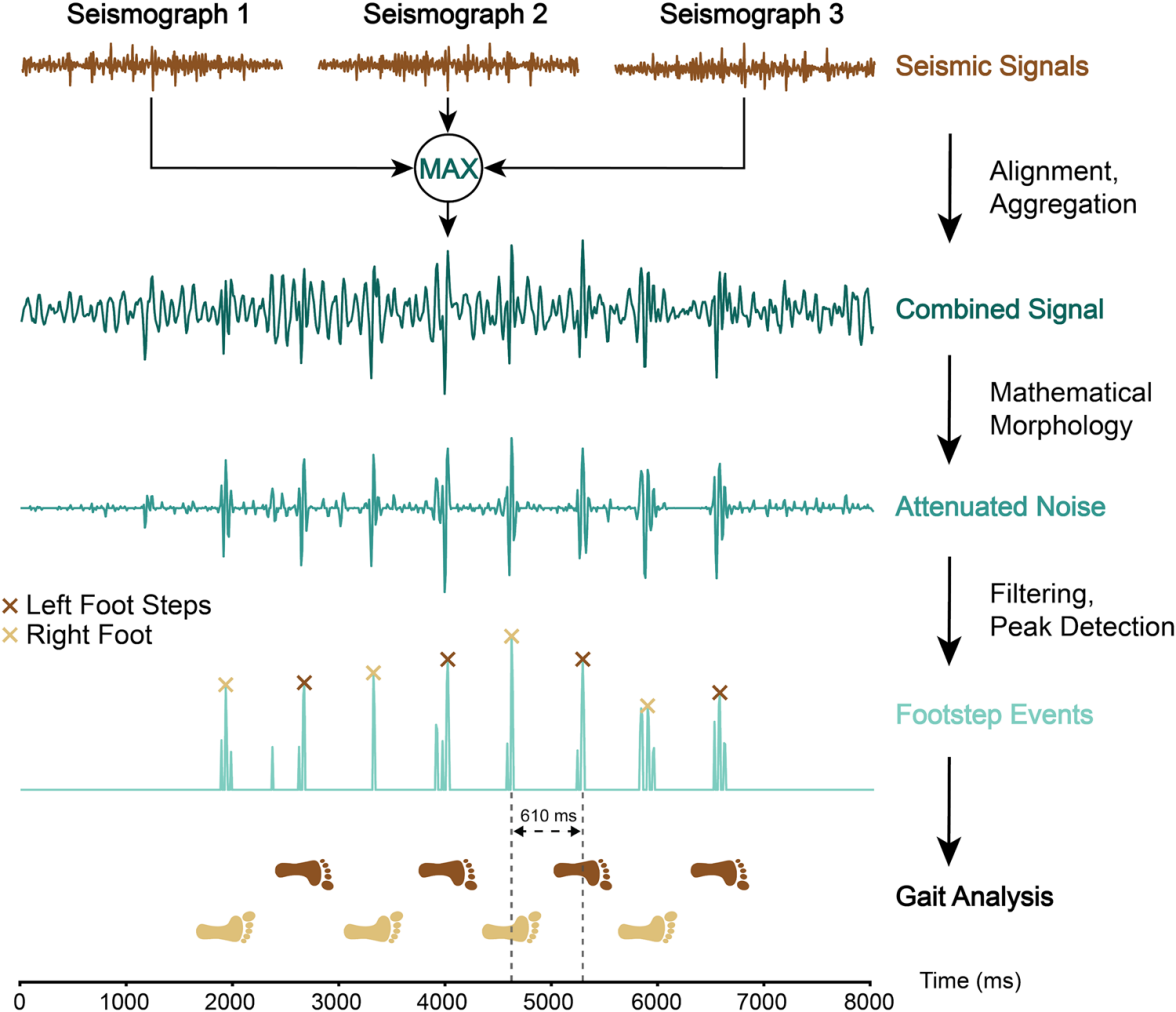
Illustration of the stages involved in the seismograph-based step extraction algorithm developed to analyze human gait. Initially, the raw seismic signals of the three seismographs were aligned and then aggregated using a rolling maximum window. This composite signal underwent a process involving a mathematical morphological operator to mitigate noise interference. Next, a peak detection algorithm was applied to the filtered signal to identify footstep events. The timestamps of these identified footstep events served as a key input for the subsequent gait analysis.

The fundamental principle of the developed algorithm originates from the premise that human footsteps can be likened to minor earthquakes, creating vibrations that propagate through the ground and can be detected by seismographs. Hence, identifying footsteps in seismic signals corresponds to determining timestamps of peaks that resemble earthquake seismic events. The particular stages of our seismograph-based step extraction algorithm involved signal aggregation, mathematical morphology, filtering, and step detection (Fig. 5).

Initially, the measurements of the three seismographs were normalized and detrended by subtracting their mean values. The normalized signals were then temporally aligned and combined by applying a rolling maximum across their signals to enhance their overall quality. Such a maximum signal aggregation also has a positive influence on damping amplitude as it corrects degraded amplitudes, counteracting potential signal damping influences (Supplementary Material, Section S5).

A nonlinear reduction method based on mathematical morphologies was used to mitigate low-frequency noise in the seismographic signals. This type of noise attenuation can effectively identify and eliminate outliers, as well as extract key features, such as local peaks in time-series data^25,42,43^. To attenuate the signal’s noise, the average of its opening and closing was derived and then subtracted from the signal, which directly corresponds to the definition of the compound top-hat filters (CTF) (Supplementary Material, Equation S5). The choice to employ Mathematical morphologies alongside CTF was grounded in their proven effectiveness in reducing low-frequency noise in microseismic measurements while still retaining significant seismic events, such as the footstep events targeted for extraction in our study, as demonstrated by Li et al.^25^.

The opening operation was implemented by first executing an erosion on the time series, then proceeding with a dilation, which helps remove sharp spikes in the data. Conversely, the closing operation was implemented by initially performing a dilation on the time series, followed by erosion, which helps to fill in sharp dips in the data. In these computations, a spherical structural element (SE) was used to dilate and erode the signal. The radius of our SE was hard-coded to 200 ms because this value corresponds to the duration of an average single footstep during human walking^22^.

The resulting noise-attenuated signal was processed using two linear filters: a Hamming filter to boost peaks while simultaneously smoothing and a rectangular filter to eliminate potential closely adjoining double peaks. Both filters were employed using a 100 ms radius. Footsteps were extracted by applying a thresholding-based peak detector to the filtered signal. Consequently, footstep events correspond to the timing of initial contacts of the foot (i.e., the timestamp of the extracted peak), which were used in the gait parameter calculation. The robustness of the peak detector was further increased by imposing the following constraints: requiring a minimum distance of 50 ms between two peaks and anticipating that the height of any potential peak would reside within the upper 95th percentile of the min-max normalized amplitudes. No additional amplitude enhancement procedures were performed to identify peaks, as our noise-attenuated signals exhibited distinct characteristics (Supplementary Material, Section S5).

In the gait analysis, the times of the extracted footstep events per ambulation were used to compute four temporal parameters (step time, cycle time, ambulation time, and cadence), one spatiotemporal parameter (velocity), and one spatial parameter (step length) (Table 4). Concerning the methodology, the temporal parameters were calculated by exact computations, whereas the spatial parameters were determined by approximations by exploiting the knowledge of the walkway length. A mathematical model and corresponding derivations are provided in the supplementary material (Supplementary Material, Section S3). This model estimates the location of seismic events of interest (e.g., steps) by comparing the time differences of event arrivals between time-synchronized stationary seismographs. To realize such a comparison, the seismographs must be positioned to span an area enclosing the seismic events to be measured.

The corresponding approximated correlation results are presented in a separate figure (Supplementary Material, Fig. S2) so that they cannot be confused with the exact calculation results. It is also important to note that left- or right-footedness was not automatically determined but assigned for the first foot based on the camera recording.

To extract the timings of the start and end of the TUG test, a visual observation of the seismic signal combined with the camera recordings was used to confirm that the initial peak of the seismic signal corresponds to the start of the TUG test and the last peak to the end of the test. As such, the same algorithm used for the gait analysis can be applied to extract the timings of the TUG test.

The source code of our gait computation algorithm is freely available on GitHub, along with detailed documentation and a gait dataset^24^.

**Table 4:** A list of the temporal and spatial gait parameters and how they were computed in the gait analysis of the measured walk.

Gait Parameter	Computation (per Ambulation)	
	Type	Method
Step Time (s)	Exact	The average time difference of initial contact events between two steps of contralateral legs
Cycle Time (s)	Exact	The average difference in time between two steps of ipsilateral legs
Ambulation Time (s)	Exact	The sum of step times
Cadence (min^−1^)	Exact	The ratio of the number of identified steps to the ambulation time
Velocity (cm/s)	Approximated	The ratio of the walkway length to the ambulation time
Step Length (cm)	Approximated	Average of the velocity multiplied by the step time differences

The gait parameters were determined by analyzing the timing of footstep events.

### Statistical analysis

A comprehensive statistical comparison was conducted between the gait parameters computed using our seismograph-based method and the measurements acquired from the reference system (i.e., the pressure-sensitive walkway). More specifically, the two measurement systems were analyzed by comparing the calculated gait parameters from four different ambulation experiments per participant (i.e., Free Walk 1, Free Walk 2, Fast Walk, and Normal Walk). To establish an adequate comparison, we exclusively confined the statistical analysis to those seismographic measurements that occurred within the time frame of the walkway recordings.

Initially, descriptive statistics of all gait parameters were calculated for the seismographs and the walkway. Scatter plots of the distributions in step lengths and velocities were qualitatively compared between the two measurement methods.

Subsequently, the presence of a significant difference between the seismograph and the walkway measurement methods was evaluated by performing paired two-sample t-tests for all matching gait parameters. We hypothesized that the mean values of matching gait parameters between the seismograph and walkway measurements were not significantly different (significance level alpha = 0.05).

Next, simple linear regression was applied to the mean values of the gait parameters from all ambulation experiments to analyze the relationship between the two measurement systems. The necessity of a linear regression model was validated by performing an F-test. This test incorporated the residual sum of squares for each paired set of measured and computed gait parameters. The coefficient of determination, $R^2$, was calculated for each matching gait parameter to help clarify the error of the linear models. The correlation between the seismographic measurements and the walkway measurements of matching gait parameters was quantified by computing the Pearson correlation coefficient, r. To qualitatively interpret the correlation results, we applied the terminology proposed by Schober et al ^44^.

Finally, measured matching gait parameters were qualitatively examined using Bland-Altman plots. These plots were used to identify the presence of proportional and constant biases in the data within the bounds of the confidence intervals, as well as to determine the agreements between the matching gait parameters measured by the walkway and those measured by the seismographs.

The same statistical analysis procedures and visualization techniques were applied to report the results of our TUG test estimations and the ground-truth timings extracted from the video recordings. Our second hypothesis stated that the mean values of the TUG times between the seismograph and camera system were not significantly different.

### Supplementary Information

Supplementary Information 1.

## Data Availability

The raw data that support the findings of this study are not openly available due to reasons of sensitivity. The associated processed raw data is available from the corresponding author, M.S., upon reasonable request.
