# Supplementary material for: Unobtrusive measurement of gait parameters using seismographs: An observational study
Source: Sci Rep. 2024 Jun 24;14:14487. doi: 10.1038/s41598-024-64508-4 (PMC11196696; doi:10.1038/s41598-024-64508-4)
Supplement: Supplementary file 1 — Supplementary Information 1. [file 41598_2024_64508_MOESM1_ESM.pdf]

Unobtrusive Measurement of Gait Parameters Using Seismographs: An Observational Study

Michael Single[1,*], Lena C. Bruhin[1], Aileen C. Naef[1], Paul Krack[2], Tobias Nef[1,2], and Stephan M. Gerber[1]

[1]Gerontechnology and Rehabilitation Group, ARTORG Center for Biomedical Engineering Research, University of Bern, Bern, Switzerland.
[2]Department of Neurology, Inselspital, Bern University Hospital, University of Bern, Bern, Switzerland
[*]email: michael.single@unibe.ch

ABSTRACT

Gait analysis helps to detect human locomotion abnormalities that can signal health changes. Traditional observation-based assessments have limitations due to subjective biases and capture only a single time point. Ambient and wearable sensor technologies allow continuous and objective locomotion analysis, yet face challenges due to the need for specialized expertise and user compliance. This study presents a seismograph-based method for quantifying human gait, aiming to match the accuracy of a clinical reference measurement system. We developed a step extraction algorithm based on mathematical morphologies to realize seismograph-based gait analysis. Clinical gait parameters of 50 healthy individuals were measured using seismographs and reference systems (pressure-sensitive walkway and video recording system). Each participant undertook four predefined ambulation experiments by walking over the walkway and performing the timed up-and-go (TUG) test. We observed linear relationships with strong positive correlations (R^2 > 0.9) between gait parameters (step time, cycle time, ambulation time, and cadence) measured with the seismographs and the pressure-sensitive walkway reference system. Moreover, the lower and upper 95% confidence intervals of all gait parameters were high. The developed algorithm can accurately derive clinical gait parameters from seismographic signals and TUG mobility test timings that did not significantly differ from reference systems.

S1 Morphological Filtration for Time Series

Mathematical morphology (MM) is a filtering technique commonly employed in computer vision to extract the geometric features of objects contained in digital images[1]. In the context of signal analysis, MM has been adapted for time series to study signal structures and extract features[2]. The basic idea of morphological filtering involves processing a signal f using a structural element (SE) g, a basic shape that resembles the features of interest contained in f. This filtering is achieved by relying on two fundamental operators, dilation ($\oplus$) and erosion ($\ominus$). Mathematically, dilating f by the SE g is defined as

$$(f \oplus g)(t) = \max\left(f(z-x) + g(x) | x \in g, (z-x) \in f\right) \tag{S1}$$

Eroding f by g is defined as

$$(f \ominus g)(t) = \min\left(f(z+x) - g(x) | x \in g, (z+x) \in f\right) \tag{S2}$$

The shape and size of the SE typically depend on the target signal f and determine the performance of the morphology-based processing. The purpose of the dilation operation is to expand or smooth peaks in the signal, whereas the erosion contracts or sharpens troughs. The compound operations, the opening ($\circ$) and the closing ($\bullet$), are defined as the sequential combination of the dilation and the erosion operations[1]. More precisely, applying an erosion followed by a dilation is denoted as the opening operation, and applying a dilation followed by an erosion is denoted as the closing operation. In terms of mathematical equations, the opening is defined as

$$f \circ g = (f \ominus g) \oplus g \tag{S3}$$

The formula of the closing is

$$f \bullet g = (f \oplus g) \ominus g \tag{S4}$$

We built upon Li et al.'s compound top-hat filter (CTF) to develop our step-extraction algorithm[3]. This filter is designed to extract large-scale information from a signal by subtracting the combined results of the opening and closing operators from the signal itself. The CTF is defined as

$$\mathrm{CTF}(f) = f - \frac{f \bullet g \circ g + f \circ g \bullet g}{2} \tag{S5}$$

A visualization of the morphological operations applied on a seismographic signal, together with the intermediate results of the CTF, is shown in Figure S1 in the form of a computation graph.

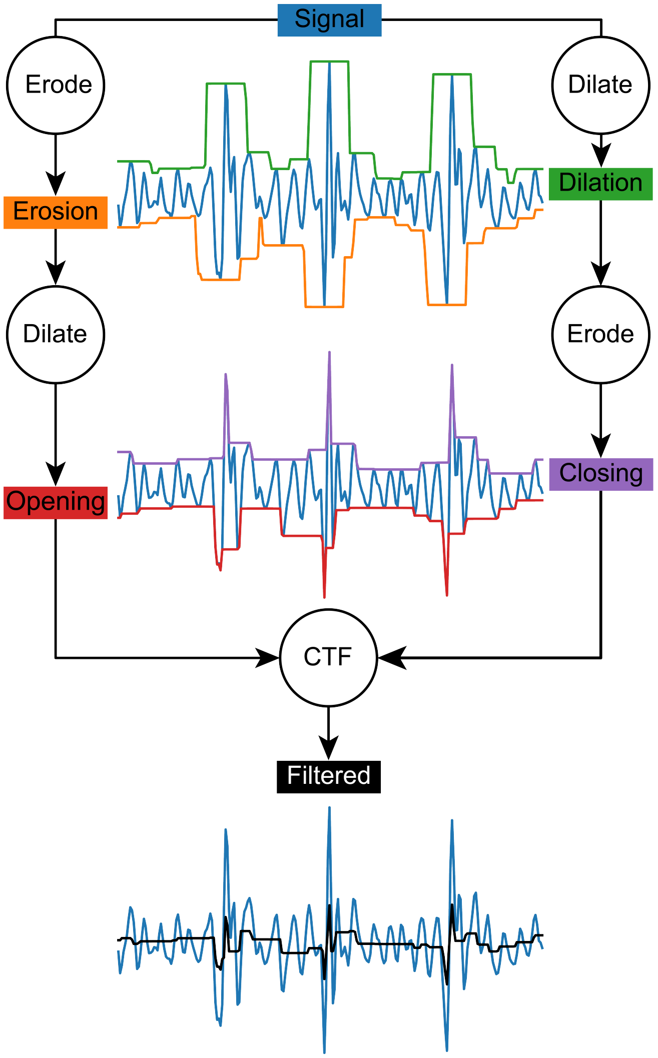

Figure S1. Illustration of the computation graph of the compound top-hat filter (CTF) together with visualizations of the intermediate filter results. The seismic signal is initially eroded (orange graph) and dilated (green graph). The opening (red graph) is computed by dilating the previously eroded result, whereas the closing (purple graph) is computed by eroding the previously dilated result. The final filtered signal (black graph) is computed by applying the CTF operator on the opening and closing. A spherical SE was used throughout the morphological operations.

S2 Correlations of Spatial Gait Parameters

The correlation of the approximated spatial gait parameters (i.e., velocity and step length) showed a strong positive correlation between measurements assessed with the seismographs and the pressure-sensitive walkway (Fig. S2).

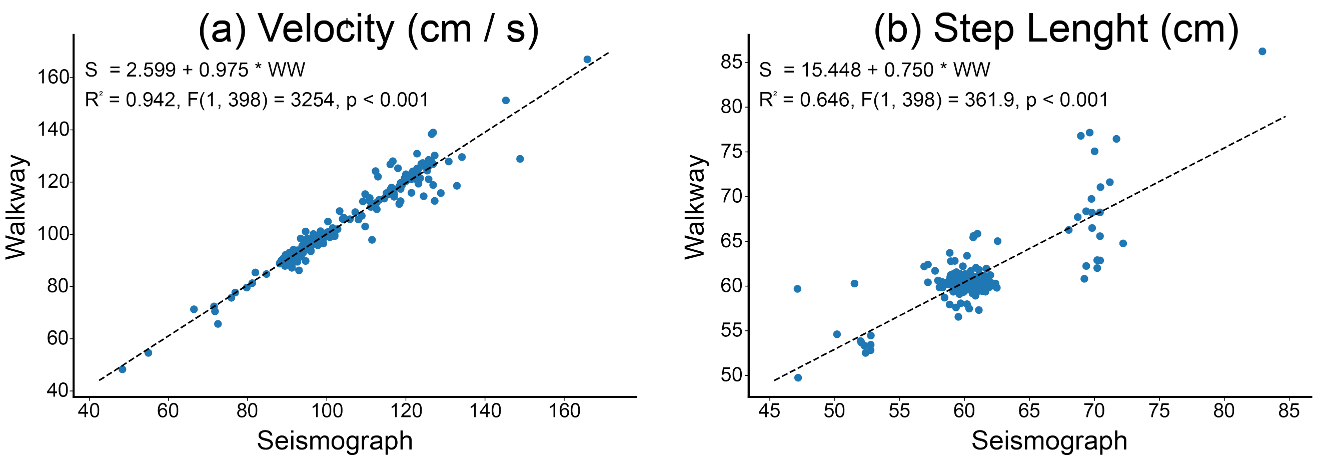

Figure S2. Plots of the linear relationships of the approximated gait parameters between measurements assessed using the pressure-sensitive walkway (WW) and the seismographs (S). In each plot, we provide the slope and inclination of the fitted linear model, the determination coefficients, and the F-scores. (a) Velocity and (b) Step Length

S3 Localization using Seismographs

To determine the location of seismic events of interest (e.g., steps), the time differences of event arrivals between time-synchronized stationary seismographs can be compared. To realize such a comparison, the seismographs must be positioned to span an area enclosing the seismic events to be measured. Figure S3 illustrates an exemplary setup of seismograph-based step-event localization.

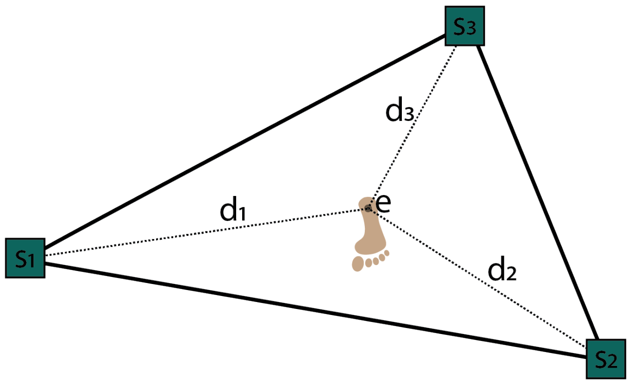

Figure S3. Illustration for determining the position of a step based on the localization of a seismic event (i.e., the initial contact of a footstep): Three time-synchronized seismographs $\mathbf{s_1}$, $\mathbf{s_2}$, and $\mathbf{s_3}$ were positioned to span a triangular area enclosing the induced seismic event $\mathbf{e}$. The distance d_k between each seismograph $\mathbf{s_k}$ and the event source $\mathbf{e}$ can be estimated by correlating the measured event time $\mathbf{t_e}$ in each sensor together with the sensor locations.

In the following, $\mathbf{s_k} = (\mathbf{x_k}, \mathbf{y_k}) \in \mathbf{R^2}$ denotes fixed location of the k-th seismograph, $\mathbf{e} = (\mathbf{x_e}, \mathbf{y_e}) \in \mathbf{R^2}$ the source location of the seismic event of interest, and $\mathbf{t_e}$ the time when the event $\mathbf{e}$ occurred. The transmission time t_k between the event source and the k-th sensor is defined as

$$t_k = t_e + \frac{d_k}{v} \tag{S6}$$

,

where $d_k = \|\mathbf{e} - \mathbf{s_k}\|_2$ denotes the Euclidean distance between the event source and the k-th sensor. By re-arranging the terms in equation S6, using the definition of the distance d_k, and using the assumption of having constant wave propagation

velocity, we can derive the following expression:

$$\begin{aligned} const \equiv v = \frac{d_k}{t_k - t_e} &= \frac{\|\mathbf{e} - \mathbf{s_k}\|_2}{t_k - t_e} \\ \implies \forall i \neq j : \frac{s_i - e}{t_i - t_e} &= \frac{s_j - e}{t_j - t_e} \\ \iff (s_i t_i - s_j t_j) &= (s_i - s_j)\, t_e + (t_i - t_j)\, e \end{aligned} \tag{S7}$$

By relying on the assumption that three seismographs s_1, s_2, and s_3 were used, equation S7 gives us the following linear system of equations

$$\underbrace{\begin{pmatrix} x_1t_1 - x_2t_2 \\ y_1t_1 - y_2t_2 \\ x_1t_1 - x_3t_3 \\ y_1t_1 - y_3t_3 \end{pmatrix}}_{\mathbf{y}} = \underbrace{\begin{pmatrix} x_1 - x_2 & t_1 - t_2 & t_1 - t_2 \\ y_1 - y_2 & t_1 - t_2 & t_1 - t_2 \\ x_1 - x_3 & t_1 - t_3 & t_1 - t_3 \\ y_1 - y_3 & t_1 - t_3 & t_1 - t_3 \end{pmatrix}}_{\mathbf{A}} \underbrace{\begin{pmatrix} t_e \\ e_x \\ e_y \end{pmatrix}}_{\mathbf{x}}. \tag{S8}$$

The location of the event $e = (e_x, e_y)$ and the time t_e when the event occurred is obtained by solving for $\mathbf{x}$ in equation *S8*

$$\mathbf{x} = \mathbf{A}^{-\mathbf{1}}\mathbf{y}. \tag{S9}$$

It is important to note that the accuracy of event localization is intrinsically linked to the temporal resolution of the measured timestamps. This resolution, in turn, hinges on the sampling rate of the sensor. Moreover, the localization approach described in Equation S7 assumes that the wave propagates at a constant velocity. However, this assumption may not always hold due to factors such as damping or variations in the materials through which the wave travels. To address the challenge of variable velocity, Mirshekari et al. introduced a maximum likelihood estimator. This solution frames the issue of non-constant velocity as an optimization problem, offering a robust method to enhance localization accuracy under these conditions[4].

S4 Timed Up and Go Assessment

To administer the Timed Up and Go (TUG) assessments, we adhered to a structured protocol[5]. Participants were asked to traverse the walkway without wearing their shoes for this assessment, thereby eliminating the dampening effect of footwear as a confounding variable. The procedure begins with the participant seated comfortably in a standard armchair, facing a clearly marked line 3 meters away on the floor. Instructions are then given to the participant as follows:

"Upon the signal 'Go,' you will be required to: 1. Stand up from the chair; 2. Walk at your natural pace to the marked 3-meter line on the floor; 3. Execute a turn; 4. Return to the chair at your natural walking speed; 5. Sit down once more."

The timing of this assessment starts with the signal "Go" and stops when the participant is seated again.

S5 Combining Seismic Signals

Figure S4 qualitatively illustrates the influence of combining multiple seismic signals and how this can help to improve the robustness of our step detection. It is important to note that the maximal peak might not always originate from the same seismograph signal. This is clearly demonstrated in the examples highlighted by stars and circles: in the case of stars, the maximum peak is derived from the red seismic signal, whereas in the circles' case, it's from the blue signal. Our method is designed to always choose the peak with the highest amplitude from the combined signals, effectively counteracting signal-damping influences. This feature significantly enhances the robustness of our step-detection algorithm.

References

1. Haralick, R. M., Sternberg, S. R. & Zhuang, X. Image analysis using mathematical morphology. *IEEE transactions on pattern analysis machine intelligence* 532–550, DOI: 10.1109/TPAMI.1987.4767941 (1987).
2. Vávra, F., Novỳ, P., Masková, H., Kotlíková, M. & Netrvalová, A. Morphological filtration for time series. In *Conference on Applied Mathematics (APLIMAT)*, 983–990 (2004).
3. Li, H., Wang, R., Cao, S., Chen, Y. & Huang, W. A method for low-frequency noise suppression based on mathematical morphology in microseismic monitoring. *Geophysics* **81**, V159–V167, DOI: 10.1190/geo2015-0222.1 (2016).

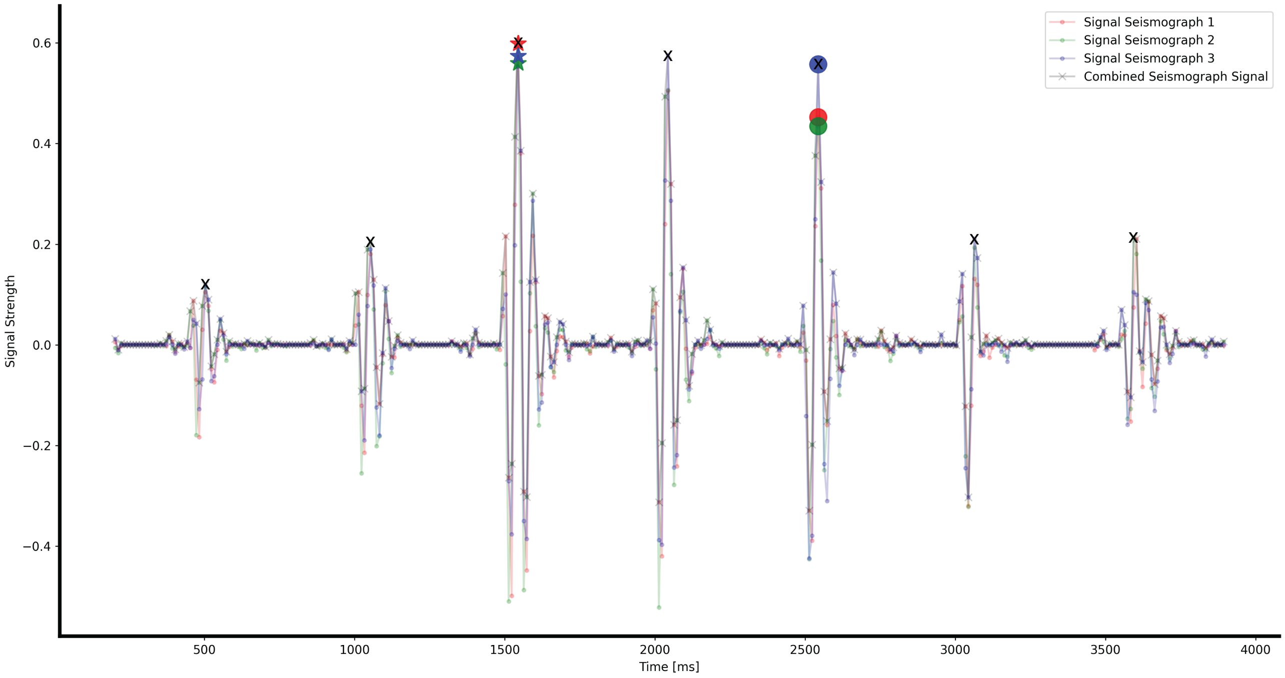

Figure S4. The diagram illustrates the dampened seismic signals depicted in red, green, and blue, alongside their aggregated signal shown in black, as processed by our newly developed algorithm that leverages mathematical morphologies and the CTF technique. The seismic activities signifying steps are marked by black crosses, where each peak in the black signal denotes the highest peak among the red, green, and blue seismic signals. Two examples illustrating how the best peaks are selected are given in this figure: First, peaks in the single seismic signals represented by star markers, and second, peaks represented as circle markers.

4. Mirshekari, M., Pan, S., Zhang, P. & Noh, H. Y. Characterizing wave propagation to improve indoor step-level person localization using floor vibration. In *Sensors and smart structures technologies for civil, mechanical, and aerospace systems 2016*, vol. 9803, 30–40, DOI: 10.1117/12.2222136 (SPIE, 2016).

5. Timed Up & Go (TUG). https://www.cdc.gov/steadi/pdf/TUG_test-print.pdf (2017). [Online; accessed 14-March-2024].
